# QTL Mapping and Candidate Gene Identification of Swollen Root Formation in Turnip

**DOI:** 10.3390/ijms22020653

**Published:** 2021-01-11

**Authors:** Yudi Wu, Shifan Zhang, Hui Zhang, Fei Li, Guoliang Li, Chuchuan Fan, Rifei Sun, Shujiang Zhang

**Affiliations:** 1 Institute of Vegetables and Flowers, Chinese Academy of Agricultural Sciences, Beijing 100081, China; 82101171118@caas.cn (Y.W.); zhangshifan@caas.cn (S.Z.); zhanghui05@caas.cn (H.Z.); lifei@caas.cn (F.L.); liguoliang@caas.cn (G.L.); 2 National Key Laboratory of Crop Genetic Improvement, Huazhong Agricultural University, Wuhan 430070, China

**Keywords:** *Brassica rapa*, turnip, swollen root, QTL, candidate gene

## Abstract

The swollen root is an important agronomic trait and is a determinant of yield for turnips, which are cultivated as both vegetables and fodder. However, the genetic mechanism of swollen root formation is poorly understood. In this study, we analyzed the F_2_ and BC_1_P_2_ populations derived from a cross between “10601” (European turnip with swollen root, *Brassica rapa* ssp. *rapifera*, AA, 2n = 2× = 20) and “10603” (Chinese cabbage with normal root, *Brassica rapa* ssp. *pekinensis*, AA, 2n = 2× = 20), and suggested that the swollen root is a quantitative trait. Two major quantitative trait loci (QTLs), *FR1.1* (Fleshy root 1.1) and *FR7.1* (Fleshy root 7.1), were identified by QTL-seq analysis and further confirmed by QTL mapping in F_2_ and BC_1_P_2_ populations. The QTL *FR1.1* with a likelihood of odd (LOD) of 7.01 explained 17.2% of the total phenotypic variations for root diameter and the QTL *FR7.1* explained 23.0% (LOD = 9.38) and 31.0% (LOD = 13.27) of the total phenotypic variations in root diameter and root weight, respectively. After a recombinant screening, the major QTL *FR7.1* was further narrowed down to a 220 kb region containing 47 putative genes. A candidate gene, *Bra003652*, which is a homolog of *AT1G78240* that plays an essential role in cell adhesion and disorganized tumor-like formation in *Arabidopsis thaliana*, was identified in this region. In addition, expression and parental allele analysis supported that *Bra003652* was a possible candidate gene of QTL *FR7.1* for swollen root formation in turnip. Our research may provide new insight into the molecular mechanism of swollen root formation in root crops.

## 1. Introduction

Turnip (*Brassica rapa* ssp. *rapifera*, AA, 2n = 2× = 20), which is cultivated for use as a vegetable, medicine, and fodder, is characterized by a swollen root. The turnip represents an important type in *B. rapa*, which displays considerable morphological diversity (leafy vegetables, oil crop, and stem/root crop). The swollen root is an important storage organ, which contains sugar, proteins, minerals, and many kinds of vitamins, and determines the yield and quality of turnip. Therefore, understanding the molecular mechanism underlying swollen root formation is important for improving root crops performance. Some researchers have shown that the swollen root of turnip is a taproot [1,2,3], while a few studies have mentioned that the thickened part of turnips consists of both hypocotyl and root [4,5,6]. Anatomical observation has identified that morphological changes of the xylem lead to an initiation of swollen root formation [6]. However, the genetic mechanism underlying swollen root formation and its regulation remains unknown.

With respect to turnips, the root shape and size are quantitative traits [7]. A previous study has revealed that a major quantitative trait loci (QTL), *TuQTL-1* near *BrFLC2* (Flowering Locus C), and two novel QTLs on chromosomes A1 and A5 are involved in swollen root formation [8]. In addition, 18 QTLs controlling the traits of fleshy roots, including seven QTLs for root width, five QTLs for root length, and six QTLs for root weight, have been identified based on an F_2_ population [3]. However, no swollen root-related QTLs have been fine-mapped thus far, and this makes it difficult to perform for further research.

The de novo assembly of the Chinese cabbage (Chiifu-401-42) [9] genome greatly promotes the development of functional genomics of Chinese cabbage and other *Brassica* crops. More and more genomic and transcriptomic studies have been used to study the swollen root traits [10]. The analysis of 199 *B. rapa* and 119 *B. oleracea* accessions has identified a large number of genes related to the synthesis, transport, or binding of sugar or cellulose, the expansion gene family, root development, and cell division, which participates in the formation of the storage root in turnip [11]. High-throughput analysis of small ribonucleic acid (RNA) sequences has indicated that various microRNAs may play important roles in the growth, development, and response to dark environments of turnip [12]. Comparative transcriptomics has revealed 841 differentially expressed genes (DEGs), which are homologues in turnips and kohlrabi [13]. The *Bra-FLOR1* and *Bra-CYP735A2* genes are involved in tuber initiation and/or tuber growth based on cytological, physiological, genetic, and transcriptomic approaches [6]. However, the function of these genes and microRNAs needs further verification.

The genetics underlying storage organ formation in many other crops can be used to provide a reference for turnip analysis. Species with storage organs, including radish, potato, rutabaga, carrot, beets, sweet potato, and cassava, play significant roles in agriculture, and their storage organs are controlled by complex interactions involving genetic, environmental, and physiological factors [5,6,14]. Sucrose is crucial in the development of storage organs in many root/stem crops [15,16]. The sucrose metabolism pathways are the most significantly activated in radish storage roots, and the expression levels of sucrose synthase 1 (*SUS1*) are correlated with root thickening rates [14,17], which is consistent with the study that sucrose synthase is a major enzyme involved in the storage root development of radish [16]. In addition, plant hormones, such as cytokinin, auxin, jasmonic acid (JA), abscisic acid (ABA), and gibberellin (GA) are important regulatory factors in the formation and thickening of tuberous roots [18,19,20,21,22]. Furthermore, short days and cool temperatures promote tuber formation in potato [23,24,25]. 

In this study, we observed the development and inheritance pattern of the swollen root traits in the progeny of a cross between two subspecies of *B. rapa*, European turnip with swollen root and Chinese cabbage with normal root. QTL-seq technology was used to sequence the deoxyribonucleic acid (DNA) samples from large root and small root pools and to rapidly identify major QTLs associated with swollen root traits. Then, two major QTLs were confirmed and fine-mapped using classical linkage mapping. Expression pattern analyses and comparative genomics were conducted for the candidate genes. Our research provides some candidate genes and lays a foundation for studying the molecular mechanism of swollen root formation.

## 2. Results

### 2.1. Inheritance of Swollen Root in B. rapa

**Figure 1:**
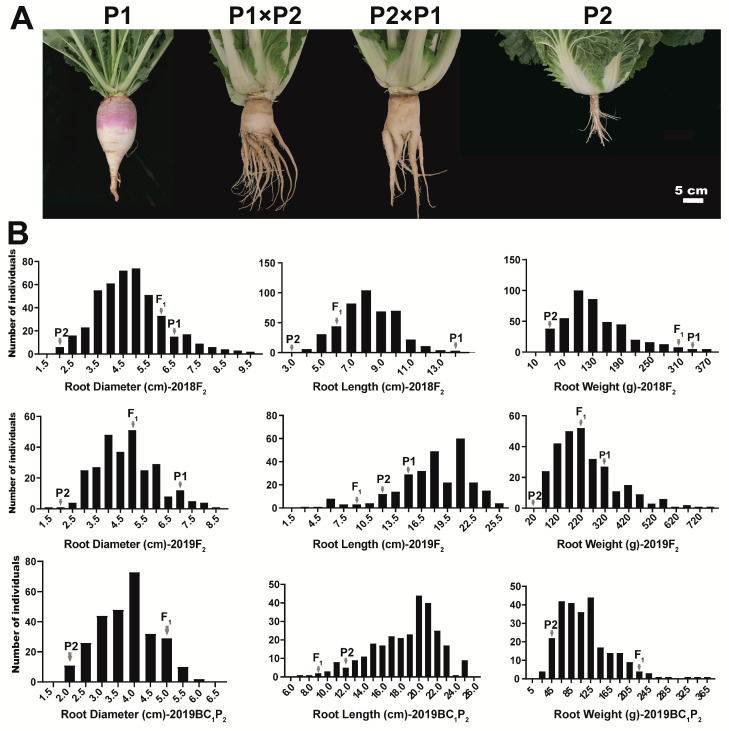
Phenotype of the parents, F_1_, F_2_, and BC_1_P_2_ populations. (**A**) Phenotype of P1 (European turnip, *Brassica rapa* ssp. *rapifera*) with swollen root, P2 (Chinese cabbage, *Brassica rapa* ssp. *pekinensis*) with normal root, and two F_1_ hybrids, 90 day old plants. Bar, 5 cm; (**B**) Frequency distribution of root diameter, root length, and root weight in F_2_ and BC_1_P_2_ populations, in 2018 and 2019.

Significant differences in root-related traits were observed among the “10601” (P1, swollen root), “10603” (P2, normal root), and F_1_ hybrids, in 2018 and 2019 (Figure 1A and Table 1). P1 exhibited a larger swollen root than P2, with an average root diameter of 6.4–7.0 cm, root weight of 316.0–330.0 g, and root length of 13.5–14.1 cm; P2 had a small root with an average root diameter of 1.5–1.9 cm, root weight of 23.8–30.8 g, and root length of 2.5–12.0 cm, in 2018 and 2019 (Table 1). The value for the F_1_ hybrid (except root length in 2019) was intermediate between those of its two parents, but much closer to that of P1 (Table 1). F_1_ plants derived from reciprocal crosses between P1 and P2 exhibited obvious swollen roots, which suggested that the swollen root trait was dominant in this cross (Figure 1A). 

The root diameter, root weight, and root length traits showed large coefficients of variation (CV%) among the F_2_ and BC_1_P_2_ populations, and continuous distribution in three segregating populations (F_2_ in 2018, F_2_ in 2019, and BC_1_P_2_ in 2019), which indicated that the root-related traits are quantitative in *B. rapa* (Figure 1B and Table 1).

### 2.2. Phenotypic and Anatomical Observation of the Hypocotyl and Root from Parents

**Figure 2:**
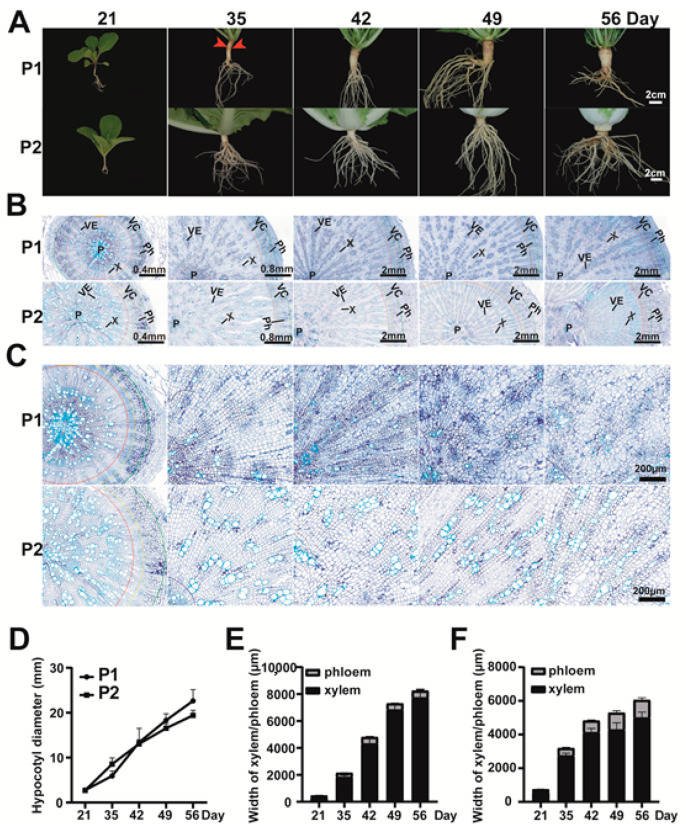
Phenotypic and cytological observation of P1 and P2. (**A**) Phenotypic observation of the root and hypocotyl of P1 (turnip) and P2 (Chinese cabbage) at 21, 35, 42, 49, and 56 days after germination (DAG). Red arrows represent the cortex splitting phenotype. Three biological replicates were observed for each time point. Bar, 2 cm; (**B**) Bottom part of hypocotyls from P1 and P2 at 21, 35, 42, 49, and 54 DAG. P, pith; VE, vessel; X, xylem; Ph, phloem; VC, vascular cambium; (**C**) Xylem cells of P1 and P2 at 21, 35, 42, 49, and 54 DAG. Bar, 200 µm; (**D**) The hypocotyl diameter of P1 and P2 plants in different development stages; (**E**,**F**) The xylem width and phloem width of hypocotyls from P1 (**E**) and P2 (**F**).

Phenotypic observation of parent plants in different development stages showed that the swollen root of turnip consisted of hypocotyl and root tissues (Figure 2). The diameter of the hypocotyl showed an increasing trend in P1 and P2 from 21 to 49 days after germination (DAG) (Figure 2A,D). The root had an obvious cortex splitting phenotype on 35 DAG (Figure 2A, red arrow) and the root diameter exhibited a significant increase from 35 DAG in P1 plants, which indicated that the cortex splitting stage was also a sign of a transition from primary growth to secondary growth in turnip, similar to radish [17,26,27]. 

The bottom part of hypocotyls from the two parents was selected for the anatomical sections, and we found that the phloem (Ph), vascular cambium (VC), xylem (X), vessel (VE), and pith (P) could be clearly distinguished among the five developmental stages (Figure 2B). From 21 to 35 DAG, the xylem width of P1 was narrower than that of P2. Then, the xylem width increased significantly from 35 to 56 DAG in P1, while that of P2 showed limited radial growth (Figure 2B,C,E,F). There was little difference in the phloem between P1 and P2. These results indicated that the hypocotyl of turnip strongly increased in diameter, mainly by the rapidly expanding xylem parenchyma layer.

### 2.3. Quantitative Trait Loci (QTL)-seq Predicted Candidate Regions for Controlling the Swollen Root Traits

**Figure 3:**
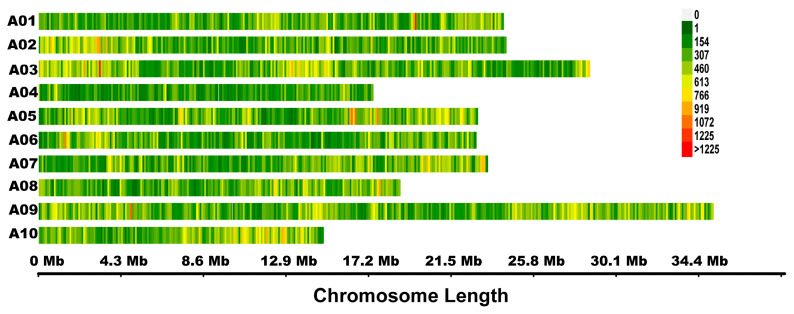
Distribution of 753,153 single nucleotide polymorphisms (SNPs) used in quantitative trait loci (QTL)-seq analysis. Different colors show the density of SNPs within a sliding window of 100 kb.

In total, 15.47–15.99 Gb clean reads were obtained for the P1, P2, L-pool, and S-pool. The average sequencing depth ranged from 29.21 to 33.86×, and the coverage ranged from 89.79 to 95.76% (Table S1). Using the *B. rapa* genome V1.5 as reference, 100,565–261,027 insertion and deletion (InDel) and 427,823–1,369,893 single nucleotide polymorphisms (SNP) variations were identified for P1, P2, L-pool, and S-pool (Table S2). Finally, 753,153 high-quality SNPs that were homozygous for each parent and polymorphic in the two parents were obtained and used for calculating the SNP-index and ∆ (SNP-index). A distribution map of the SNPs was drawn by plotting the number of SNPs using a sliding window of 100 kb (Figure 3).

**Figure 4:**
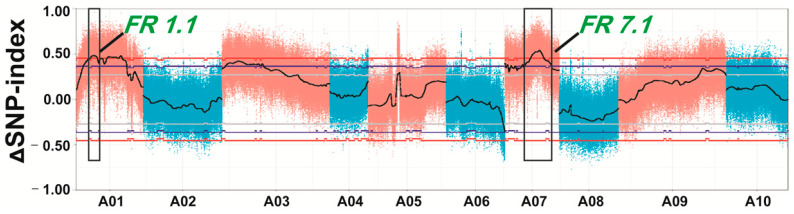
The ∆ (SNP-index) graph of two extreme pools. The black line is the average value of ∆ (SNP-index) in a sliding window of 1 Mb. Blue and red lines represent statistical confidence intervals *p* < 0.1 and *p* < 0.05, respectively. Two regions on chromosome A01 and A07 (black box) were identified.

According to the analysis of the SNP-index and ∆ (SNP-index), two candidate regions were identified; one region, *FR1.1* (fleshy root 1.1), was at 4.26–8.09 Mb on chromosome 1 and the other region named *FR7.1* (fleshy root 7.1) was at 16.01–21.99 Mb on chromosome 7 (Figure 4). The absolute values of ∆ (SNP-index) of *FR1.1* and *FR7.1* were greater than the threshold and were close to 0.47 and 0.51 at a confidence level of 95%. Observations of SNP haplotypes among the plants with large roots in the large pool (L-pool) were the same as those in the “10601” parental line, while plants with small roots in the small pool (S-pool) contained alleles from the “10603” parental line in the two regions, indicating that there may have been major QTLs controlling swollen root in these regions.

### 2.4. Confirmation and Fine-Mapping of the Two QTLs: FR1.1 and FR7.1

Traditional QTL analysis, based on 165 F_2_ plants and 153 BC_1_P_2_ plants in two environments, was performed to verify the QTLs *FR1.1* and *FR7.1* detected by QTL-seq analysis. Fifty-two pairs of InDel markers and 39 pairs of SNP markers at the target regions of the two *QTLs* were designed, based on the whole genome sequencing data of the parents (Tables S3 and S4). 

**Figure 5:**
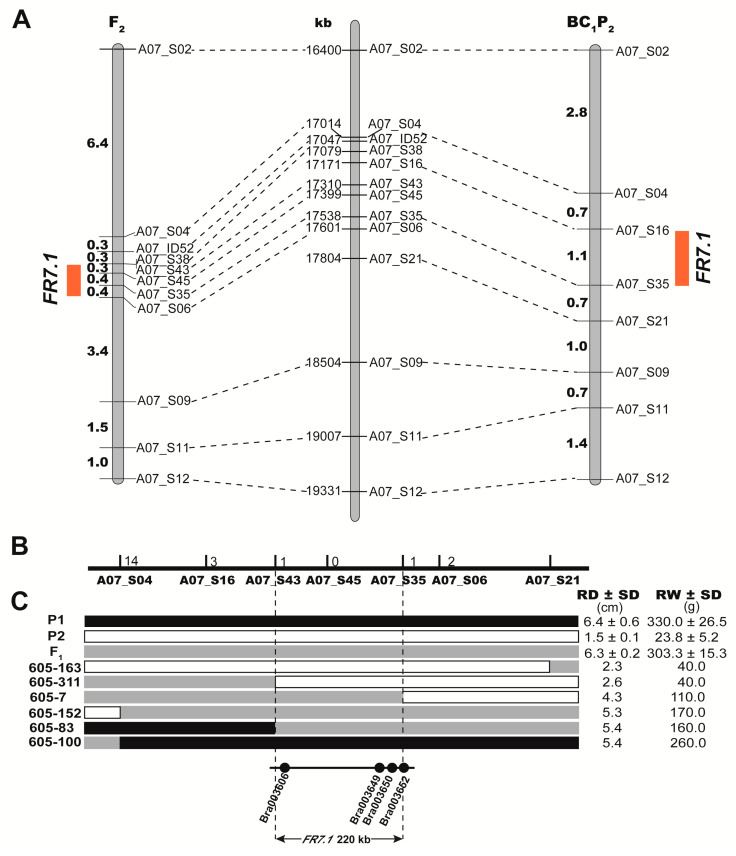
Mapping of the swollen root related quantitative trait loci (QTLs). (**A**) Locations of the *FR7.1* (Fleshy root 7.1) locus on two linkage maps. The left and right linkage maps were constructed using F_2_ and BC_1_P_2_ populations, respectively. Numbers show the genetic distances between adjacent markers (cM), the red box denotes the QTL peak position. The map located in the middle of two linkage maps is a physical map of *B. rapa* A07 chromosome in the target region of the *FR7.1* locus. Numbers show the physical distances; (**B**) Fine mapping of the *FR7.1* locus in *B. rapa*. The numbers on the black line represent the number of recombinants at the corresponding markers; (**C**) Recombinants in the position of *FR7.1* between SNP markers A07_S04 and A07_S21 (upper figure). RD: root diameter. RW: root weight. SD: standard deviation. The corresponding genes annotated according to the reference genome are presented in the 220 kb interval region (lower figure).

Among the developed markers on chromosome 7, 13 markers that covered the QTL region and showed polymorphism between parents were used to genotype the segregating population for QTL analysis. In the BC_1_P_2_ population, *FR7.1* was narrowed to the 17.01–17.80 Mb region between the SNP markers A07_S04 and A07_S21, which explained 13.10% and 16.00% of the phenotypic variation of root diameter and root weight, respectively (Table 2 and Figure 5A). In the F_2_ population, *FR7.1* was located within the 17.31–17.60 Mb region between the SNP markers A07_S43 and A07_S06 and explained 23.00% and 31.00% of the phenotypic variation of root diameter and root weight with likelihood of odd (LOD) values of 9.38 and 13.27, respectively (Table 2 and Figure 5A). By combining the QTL mapping results from the F_2_ and BC_1_P_2_ populations, the QTL *FR7.1* was confirmed and mapped to the marker interval between A07_S43 (17.31 Mb) and A07_S06 (17.60 Mb) on chromosome 7. After a recombinant screening using 716 plants from F_2_-2018, F_2_-2019, and BC_1_P_2_-2019 populations, the QTL *FR7.1* was further narrowed down to a 220 kb region (17.31–17.53 Mb) bounded by A07_S43 and A07_S35 (Figure 5B,C).

Similarly, 10 polymorphic InDel markers that covered the *FR1.1* region were used for linkage analysis in the F_2_ population. A major QTL for root diameter between two InDel markers, A01_ID03 and A01_ID31, was identified by QTL mapping analysis, which was physically located in the region of 4.62–5.29 Mb on chromosome 1. This QTL explained 17.2% of the phenotypic variation in root diameter with a LOD of 7.01 (Table 3). Thus, *FR1.1* was also confirmed and narrowed down to a 670 kb region (4.62–5.29 Mb) on chromosome 1.

### 2.5. Identification of Candidate Genes for FR7.1 in Turnip

Based on the results of QTL-seq and QTL mapping, *FR7.1* was selected for further analysis. The candidate interval contained 47 predicted genes in the *B. rapa* reference genome (http://brassicadb.org/brad/index.php). Among these genes, 31 genes have SNP variation between the two parents (Table S5). According to the current research, sucrose metabolism, hormonal and the cell cycle pathways are the central components of tuberization in radish, potato, and *B. juncea* [6,17,28,29]. Therefore, four genes involved in the cell cycle, glycolysis/gluconeogenesis, and plant hormone signal transduction were selected, namely, *Bra003606*, *Bra003649*, *Bra003650*, and *Bra003652*, according to the annotations (Figure 5C and Table S6).

**Figure 6:**
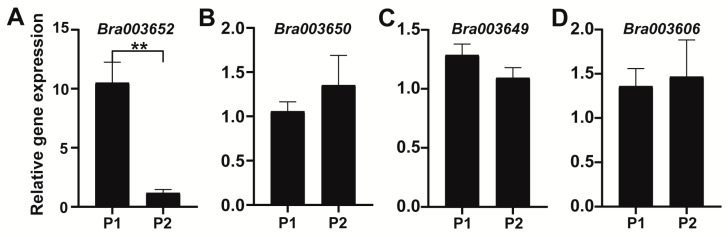
The expression patterns of candidate genes in the hypocotyl from P1 (turnip) and P2 (Chinese cabbage). Values are the mean ± SD of three biological replicates. ** significantly different at *p* < 0.01.

In order to determine whether the expression levels of the candidate genes may contribute to the variance of swollen root traits, we studied the expression patterns of *Bra003606*, *Bra003649*, *Bra003650,* and *Bra003652* in the hypocotyls of P1 and P2 via quantitative real-time polymerase chain reaction (qRT-PCR). The results showed that the expression level of *Bra003652* was significantly higher in P1 than in P2 (*p* < 0.01). However, the expression levels of *Bra003606*, *Bra003649, and Bra003650* did not differ between P1 and P2 (Figure 6). Therefore, *Bra003652* aroused our attention.

The physical location of the *Bra003652* gene is 17,537,326-17,540,584 bp, which contains the SNP marker A07_S35 at which the LOD value curve peaked for *FR7.1* (Figure 5C, Table 2 and Table S6). *Bra003652* is a homolog of *AT1G78240*, which encodes the tumorous shoot development 2 (TSD2) protein and is related to root development in *Arabidopsis thaliana* [30]. *Bra003652* and *AT1G78240* are 85.38% identical at the nucleotide level (Figure S1) and share 86.14% amino acid identity (Figure S2). This suggests that these genes encode enzymes with similar functions.

**Figure 7:**
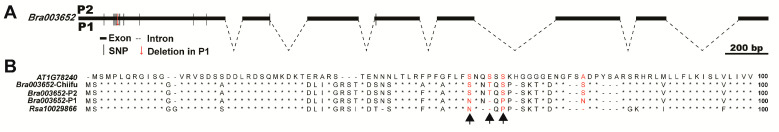
Analysis of genes structure and alignment of protein sequences. (**A**) The *Bra003652* gene structure and natural variations between the alleles from P2 and P1; (**B**) Alignment of protein sequences among homologous genes from *Arabidopsis*, Chiifu (Chinese cabbage), P2 (Chinese cabbage), P1 (turnip), and radish. Red numbers indicate the positions of the amino acid variations between P2 and P1. Black arrows indicate the variations that were the same between *Bra003652*-P1 and *Rsa10029866*.

To define the structure of *Bra003652*, the amplified products of the complementary DNA (cDNA) and genomic DNA (gDNA) fragments were sequenced using *Bra003652*-specific primers. The result showed that *Bra003652* contains eight exons and seven introns, encoding a protein of 684 amino acids (Figure 7A and Figure S2). The putative protein contains a putative S-adenosyl-L-methionine-dependent methyltransferase domain (Figure S2). Comparative sequence analysis was used to align the genomic DNA sequences of *Bra003652* from both parents. Some variations were detected in the open reading frame of *Bra003652*, including thirteen SNP variations and a three-base deletion between the two parents (Figure 7A). A multiple protein sequence alignment of *Bra003652* and its homologous genes from five inbred lines, including two materials with swollen root (P1 and radish) and three materials with normal root (*Arabidopsis*, Chiifu, and P2), was conducted. There were three amino acid variations and one amino acid deletion between P1 and P2, which did not occur in the region of the putative domain (Figure 7B and Figure S2). Among the four variations, the first three variations (S → N, an amino acid deletion and S → P) were the same for P1 and radish, and they were different among *Arabidopsis*, Chiifu, and P2 (Figure 7B, black arrows). This gave rise to a hypothesis that these variations might influence the function of *Bra003652* and involve in swollen root formation in turnips.

At the same time, we also analyzed the sequences of *Bra003606*, *Bra003649,* and *Bra003650* from parents. Some variations were found in the coding sequences of *Bra003649* and *Bra003606* (Figures S3 and S5), while that of parents were identical in *Bra003650*. The putative protein sequences of parents were identical in *Bra003649* (Figure S4), while an amino acid variation was found between P1 and P2 in *Bra003606* (Figure S6). Further comparative analysis showed that the mutation type of this amino acid was consistent in P1 (turnip) and Chiifu (Chinese cabbage), but different from P2 (Chinese cabbage), which indicated that the variation of *Bra003606* might not be involved in the formation of swollen root (Figure S6). These results further support that *Bra003652* is the most possible candidate gene in *FR7.1*.

## 3. Discussion

### 3.1. QTL-seq Technology Accelerated the Research of Root-Related Traits

Genetic analysis shows that most root-related traits are quantitative traits controlled by multiple genes [3,7]. In our study, a European turnip and a Chinese cabbage were selected as the parental lines and used to construct the F_1_, F_2_, and BC_1_P_2_ populations. Genetic analysis showed that these traits exhibited quantitative inheritance patterns, consistent with the previous research results (Figure 1).

Many QTLs related to root traits have been identified and are scattered on multiple chromosomes in turnip, but a few candidate genes have been identified [3,7,8]. Traditional QTL mapping includes primary mapping, fine mapping, and the identification of candidate genes, which are time-consuming and expensive. QTL-seq technology, which has been applied to the QTL mapping of many traits in different species [31,32,33], was used and identified two QTLs, *FR7.1* and *FR1.1*, in this research (Figure 4). Subsequently, traditional QTL analysis of the F_2_ and BC_1_P_2_ populations was used to verify the accuracy of QTL-seq analysis. InDel and SNP markers were developed and used to construct linkage maps in segregated populations. InDel is widely used in traditional QTL mapping by polyacrylamide gel electrophoresis (PAGE) or agarose gel electrophoresis technology, which is accurate but time-consuming and labor-intensive. With the development of next-generation sequencing technologies, the genome of *B. rapa* was sequenced and assembled in 2011 [9]. Subsequently, many materials with different ecological types have been sequenced or re-sequenced by whole genome sequencing (WGS) or specific length-amplified fragment sequencing [11,34], which has revealed a large number of SNPs among different materials. In our research, we obtained 753,153 high-quality SNPs that showed polymorphism between P1 and P2. In addition, SNPs can be genotyped by Kompetitive Allele Specific PCR (KASP), which is one of the SNP genotyping platforms and has evolved to be global benchmarking technology [35]. SNP markers were chosen to construct a local linkage map and to fine-map *FR7.1*. Then, *FR7.1* was narrowed to a 220 kb region between two SNP markers, A07_S04 and A07_S35 (Table 2 and Figure 5), consistent with the result of QTL-seq analysis. The results showed that the method of combining QTL-seq and QTL mapping can rapidly and accurately identify QTL.

### 3.2. The Authenticity and Reliability of FR1.1 and FR7.1

Previously, Cheng has shown that the domestication of tuber-forming morphotypes was the result of parallel selection and convergent crop domestication and identified 24 and 26 genomic regions under selection in turnip using reduction of diversity (ROD) and PiHS population-based integrated haplotype score (PiHS), respectively [11]. Some of these identified genomic regions have overlap with the target regions of *FR1.1* and *FR7.1*, which further proves the reliability of *FR1.1* and *FR7.1*. However, the two QTLs have not been reported by other researchers through traditional genetics and may be the new loci involved in the formation of swollen root.

In our study, *FR7.1* was narrowed to 220 kb between two SNP markers, A07_S45 and A07_S35, which explained 23% and 31% of the phenotypic variation in root diameter and root weight in the F_2_ population in 2018, respectively (Table 2). Then, we used the F_2_ population and BC_1_P_2_ population to verify the authenticity of *FR7.1,* in 2019. However, we were unable to scan any QTL in the 16.01–21.99 Mb on chromosome 7 in the F_2_ population (data not shown). In 2019, many plants from F_2_ populations were infected with clubroot or soft rot diseases, which affected the measurements of root-related traits. Fortunately, *FR7.1* was localized to a 790 kb physical interval by the two markers A07_S04 and A07_S21, contributing 13.1% and 16% of the phenotypic variation in root diameter and root weight, respectively, in the BC_1_P_2_ populations, which overlapped with the result of QTL mapping from 2018 (Table 2 and Figure 5). The result showed the QTL *FR7.1* related to root diameter and root weight is reliable and could be detected in 2018 and 2019.

We also narrowed the region of the *FR1.1* locus to 670 kb between A01_ID03 and A01_ID31. *FR1.1* explained 17.2% of phenotypic variation in root diameter in the F_2_ population, in 2018 (Table 3). The locus needed to be further verified and fine-mapped using new segregating populations, which might provide new genetic resources for the research of swollen root formation.

### 3.3. Analysis and Determination of Candidate Genes

In total, 47 genes were identified in the candidate region of *FR7.1*. On the basis of the Previous studies shows that plant hormones and sucrose are important regulators of root formation and development [2,16,18,19,36]. In addition, environmental factors including short sunshine and low temperatures can also promote tuber formation in potato [23,24,25]. Among 47 genes, four genes involved in the cell cycle, glycolysis/gluconeogenesis, plant hormone signal transduction, and root development were identified, namely *Bra003606*, *Bra003649*, *Bra003650*, and *Bra003652* (Table S6). According to the anatomical observation, the number and size of xylem cells influenced the width of the xylem and are related to the enlargement of the hypocotyl in turnip (Figure 2). The expression models for *Bra003606*, *Bra003649*, *Bra003650*, and *Bra003652* that were obtained via qRT-PCR support that *Bra003652* is the most likely candidate gene of *FR7.1* in *B. rapa* (Figure 6).

*Bra003652* is a homolog of *AT1G78240*, which encodes the TSD2 protein in *A. thaliana*. Mutation of the *TSD2* gene reduces cell adhesion and strongly affects individuals, resulting in non-coordinated shoot development, which leads to disorganized tumor-like growth in *A. thaliana* [30]. Some studies have shown that the *TSD* gene depended on cytokinin to negatively regulate the activity of the meristem during the development of *Arabidopsis* [34]. In rice, the *OsTSD2* regulates the root development and cellular adhesion by affecting pectin synthesis [37]. Cheng [11] has obtained a large number of genes related to the formation of swollen root in turnip, including *Bra003652*.

A multiple sequence alignment analysis identified three amino acid variations (S → N, S → P and S → N) and an amino acids deletion between P1 and P2 (Figure 7B). Among these four variations, three amino acid variations were the same in the protein sequences of *Bra003652* and *Rsa10029866* from P1 and radish (swollen root), and these were different from the variations observed in Chiifu, P2, and *Arabidopsis* (normal root, Figure 7B, black arrows). These results gave rise to a hypothesis that these variations might influence the function of *Bra003652* and be involved in the swollen root formation in turnips.

### 3.4. Root-Related Traits Positively Affect the Yield of Root Crops

Swollen root is the decisive factor for the yield of root-type crops and consists of the following three important indexes: root diameter, root length, and root weight. In addition, many above-ground traits, including plant height, plant width, and leaf weight, directly or indirectly impact the yield of root/stem crops. Correlation analysis has shown that root yield of radish, sweet potato, and carrot was positively correlated with root length and root diameter [38,39]. In our research, the correlation coefficient for root weight and root diameter was 0.71, which was higher than other correlation coefficients in the F_2_ population (Figure S7). The correlation coefficient between root weight and root length was 0.55, while the correlation coefficient between root length and root diameter was 0.47 (Figure S7). The result shows that root diameter and root length are positively correlated with root weight, which is consistent with previous conclusions. In addition, root weight is related to plant height, plant width, and leaf weight; the correlation coefficients are 0.48, 0.54 and 0.51, respectively (Figure S7). Root diameter has a greater effect on root weight than other traits from the above result. In the study, *FR1.1* explained 17.2% phenotypic variation in root diameter and *FR7.1* explained 23% phenotypic variation of root diameter, which may harbor candidate genes involved in root diameter development. Our research may provide some candidate genes and lay a foundation for improving the yield of turnip.

## 4. Plant Materials and Methods

### 4.1. Parent Materials and Population Construction

Two inbred lines, Europe turnip “10601” (P1, swollen root) and Chinese Cabbage “10603” (P2, normal root) from *B. rapa*, both were collected by the Institute of Vegetables and Flowers, Chinese Academy of Agricultural Sciences, and were used as parents in a reciprocal cross (P1 × P2 and P2 × P1) to produce F_1_ lines. F_1_ (P1 × P2) was self-pollinated and backcrossed to generate the F_2_ and BC_1_P_2_ populations. Finally, the following five populations were obtained: P1, P2, F_1_ (P1 × P2), F_1_ (P2 × P1), and F_2_ and BC_1_P_2_.

### 4.2. Phenotype Assessment

The parental line, and the F_1_ and F_2_ populations were seeded in commercial potting mix in 54 × 28 cm flats with 32-wells in a solar greenhouse on 27 July 2018. Then, the seedlings were transplanted to an experimental farm on 16 August 2018. The row spacing and plant spacing were 50 and 40 cm, respectively. On 6 August 2019, the parental lines, and the F_1_, F_2_, and BC_1_P_2_ populations were seeded directly at the experimental farm. The row spacing and plant spacing were 60 and 50 cm, respectively. In 2018 and 2019, all experiments were carried out under standard field conditions in the Institute of Vegetables and Flowers, Chinese Academy of Agricultural Sciences (Shunyi, Beijing, China), with watering and fertilizing occurring in a timely manner. Three types of fertilizer, including organic fertilizer, compound fertilizer (N/P/K = 15:15:15) (Luxi Chemical Group Co., Ltd., Liaocheng, China), and punching fertilizer (N/P/K = 30:9:12) (Linong Feng Biotechnology Co., Ltd., Beijing, China), were used in the experimental field. The organic fertilizer including cattle manure (10 m^3^/667 m^2^) and chicken manure (2 m^3^/667 m^2^) and compound fertilizer (50 kg/667 m^2^) were applied before sowing. The punching fertilizer (5–10 kg/667 m^2^) was applied five times during the growth and development of the materials.

There were 15, 15, 15, and 447 plants in P1, P2, F_1_, and F_2_ populations in 2018, respectively. In 2019, there were 15, 15, 15, 278, and 275 plants in P1, P2, F_1_, F_2_ and BC_1_P_2_, respectively. For each plant growing 90 days after germination, six traits including root diameter, root length, root weight, plant height, plant width, and leaf weight were measured and recorded with a ruler and electronic scale. The root weight was measured after cleaning and drying. The root diameter and root length data were recorded by measuring the transverse diameters and largest longitudinal length of the roots, respectively. The correlation coefficients among different traits were calculated by Pearson correlation method and drawn with the R package “corrplot”. The statistical analyses were performed using the SAS program.

### 4.3. QTL-seq Analysis

According to the phenotypic investigation of the F_2_ population in the winter of 2018, 30 individuals with extremely large roots and 30 individuals with extremely small roots were selected to build a large-root DNA pool (L-pool) and a small-root DNA pool (S-pool) by mixing the same amounts of DNA (Table S1). The genomic DNAs of “10601” and “10603” were obtained from a single plant. Genomic DNA was isolated from fresh leaves using a modified cetyltrimethyl ammonium bromide (CTAB) method [40]. The two parents and two pools were sequenced using an Illumina HiSeq 2500 instrument (San Diego, CA, USA) with 150 bp paired-end sequencing reads. The average sequence depth exceeded 30× genome coverage for each sample. The adaptor reads, unknown sequences ”N” (reads containing unknown nucleotides >10%) and low-quality reads (reads containing more than 50% bases with a *Q* value ≤ 3) were removed from the raw data using the NGSQC toolkit [41]. The clean reads from the two extreme pools and two parents were mapped to the *B. rapa* genome V1.5 (http://brassicadb.org/brad/datasets/pub/BrassicaceaeGenome/Brassica_rapa/V1.0/Bra_Chromosome_V1.5/) using Burrows-Wheeler Aligner (BWA) software with the BWA-MEM algorithm [42]. Based on the sequence alignment results, SNPs and InDels were called using Samtools and GATK (The Genome Analysis Toolkit) software. The density distribution map of the SNPs was drawn with a sliding window with a physical distance of 100 kb. Then, Annovar software was used to annotate the SNPs and InDels. Following published methods [33,43], the SNP-index for each SNP position was calculated.

The potential candidate region (*p* < 0.05) was determined by the Δ (SNP-index) curve. P2 was selected as a reference to calculate the SNP-index and Δ (SNP-index) of the two bulk pools according to the Takagi method [33]. An SNP-index = 0.5 means that both parents have an equal contribution to the bulked progeny. If an SNP locus is related to the target trait, the SNP-index of that locus will deviate significantly from 0.5. An SNP-index = 0 indicates that all short reads are from P2. Conversely, an SNP-index = 1 indicates that all short reads are from P1. Then, we calculated the average value in a 1 Mb sliding window based on the SNP-index and Δ (SNP-index) values of each SNP locus, and drew a graph showing the 95% confidence interval. If the SNPs in the two extreme pools are consistent with other traits except for the primary target region, the regions related to the target trait will show linkage disequilibrium. The area above the threshold is the area where the candidate QTL is located in.

### 4.4. Molecular Marker Development, Linkage Analysis, and QTL Mapping

To validate the QTL-seq analysis results, traditional QTL mapping was carried out. InDel markers were designed using Primer 5.0 software, and SNP markers were designed by the Laboratory of the Government Chemist based on the *B. rapa* genome v1.5. All primer pairs were synthesized by Sangon Biological and Engineering Co (Shanghai, China). Linkage analysis was performed by applying the JoinMap v4.0 [44] to the Kosambi function, and the maximum-likelihood method was used to calculate the genetic distance. MapQTL v4.0 [45] was applied to detect the QTLs using the Interval Mapping (IM) and Multiple QTL Mapping (MQM) pattern under a threshold LOD = 2.0.

### 4.5. RNA Extraction and Quantitative Real-Time PCR

In order to analyze the candidate genes related to the swollen root traits, expression patterns of the candidate genes (*Bra003606*, *Bra003649*, *Bra003650*, and *Bra003652*) were investigated by qRT-PCR. In the spring of 2020, P1 and P2 materials were seeded in the greenhouse (Haidian, Bejing, China), with temperatures ranging from 14 °C at night to 25 °C in the daytime. Six hypocotyl samples of P1 and P2 were collected at 21, 35, 42, 49, and 56 days after germination. Three samples were used for Paraffin section and the other three plants were used to harvest RNA. Total RNA was extracted using the Trizol reagent (Thermo Fisher Scientific, Shanghai, China), according to the manufacturer’s instructions. Reverse transcription was conducted by EasyScript^®^ One-Step gDNA Removal and cDNA Synthesis SuperMix (TransGen Biotech, Beijing, China), using a standard protocol. Real-time PCR was performed using ChamQ Universal SYBR qPCR Master Mix (Vazyme Biotech Co., Ltd., Nanjing, China) on an ABI QuantStudio 12K Flex real-time PCR system (Applied Biosystem, Foster City, CA, USA), according to the manufacturer’s instructions. The *Actin gene* of Chinese cabbage was used as an internal control (forward, 5′-GGAGCTGAGAGATTCCGTTG-3′, and reverse, 5′-GAACCACCACTGAGGACGAT-3′) [46]. The special primers were designed using the Primer-BLAST tool (https://www.ncbi.nlm.nih.gov/tools/primer-blast/index.cgi?LINK_LOC = BlastHome). The primer sequences are listed in Table S7. The 2^−∆∆Ct^ method [47] was used to determine the relative expression levels of the genes. Then, the average relative expression levels were calculated, and *t*-tests were performed using the SAS program to test the significance of the differences in expression levels among the different samples.

### 4.6. Histological Analysis of Hypocotyls

The hypocotyls of “10601” and “10603” plants were collected at 21, 35, 42, 49, and 54 days after germination and were fixed, embedded, cut into slices, and stained according to the conventional paraffin-sectioning methods. The slices were observed and photographed using the microscope (Nikon ECLIPSE 80i, Tokyo, Japan). The xylem and phloem width were measured with Image J software (https://imagej.en.softonic.com/).

### 4.7. Sequence Analysis of Candidate Genes

In order to clarify the difference of candidate genes between parents, the code sequences (CDS) and gene sequence were amplified from the cDNA and gDNA of P1 and P2, respectively. The open reading frame and protein sequences of *Bra003652* and *Bra003606* from Chiifu were collected, according the *B. rapa* v1.5 genome (http://brassicadb.org/brad/index.php). The protein sequence of *AT1G78240* and *Rsa10029866* which are homologous genes of *Bra003652* from *A. thaliana* and radish were obtained, according to the corresponding reference genome [48,49]. Nucleotide and protein sequences were aligned using SnapGene software, MultAlin (http://multalin.toulouse.inra.fr/multalin/multalin.html) and MUSCLE (https://www.ebi.ac.uk/Tools/msa/muscle/).

## 5. Conclusions

In summary, anatomical observation indicated that the swollen root of turnip consists of both hypocotyl and root tissues and the swollen root traits were quantitative. Two major QTLs, *FR1.1* and *FR7.1* involved in the root diameter and root weight, were detected by QTL-seq technology and classical QTL mapping in the turnip × Chinese cabbage population. The major QTL, *FR7.1*, explained 31% (LOD = 13.27) and 23.00% (LOD = 9.38) of the total phenotypic variations in root weight and root diameter, respectively. Expression pattern and comparative genomic analysis indicated that *Bra003652* was the candidate gene involved in the swollen root formation in turnips. Our research provides some candidate genes and lays a foundation for the study of the molecular mechanism of swollen root formation.

## Figures and Tables

**Table 1:** Statistical analysis of root-related traits in parents, F_1_, F_2_, and BC_1_P_2_ populations.

Trait	Year	P1	P2	F_1_	F_2_			BC_1_P_2_		
		Mean ± SE	Mean ± SE	Mean ± SE	Mean ± SE	Range	CV%	Mean ± SE	Range	CV%
RD (cm)	2018	6.4 ± 0.2 A	1.5 ± 0.1 B	6.3 ± 0.1 A	4.6 ± 0.1	1.8–11.2	30.2	−	−	−
	2019	7.0 ± 0.4 A	1.9 ± 0.1 C	4.8 ± 0.04 B	4.6 ± 0.1	1.5–8.5	25.5	3.6 ± 0.1	1.6–6.0	24.7
RW (g)	2018	330.0 ± 8.4 A	23.8 ± 1.8 B	303.3 ± 8.8 A	137.5 ± 4.3	10.0–680.0	66.8	−	−	−
	2019	316.0 ± 16.6 A	30.8 ± 2.5 C	198.0 ± 22.1 B	220.7 ± 7.8	30.0–690.0	58.6	102.2 ± 3.5	10.0–340.0	53.3
RL (cm)	2018	13.5 ± 0.5 A	2.5 ± 0.2 C	5.5 ± 0.8 B	7.7 ± 0.1	3.1–17.2	24.5	−	−	−
	2019	14.1 ± 1.5 A	12.0 ± 0.6 A	8.3 ± 0.5 B	17.2 ± 0.3	3.0–25.0	24.5	18.6 ± 0.2	8.0–25.0	19.0

RD, root diameter; RW, root weight; RL, root length. Different letters indicate a significant difference at *p* < 0.01 among means of P1, P2, and F_1._ SE, standard error. CV%: coefficients of variation (%). “−“: No data was shown.

**Table 2:** QTL mapping analysis of the *FR7.1* locus with the polymorphic markers.

Primer Name	Physical Position (bp) ^a^	F_2_-2018				BC_1_P_2_-2019			
		RD		RW		RD			RW
		LOD	%Exp ^b^	LOD	%Exp	LOD	%Exp	LOD	%Exp
A07_S04	17014037-17014237	0.08	0.20	0.08	0.20	0.10	0.30	0.95	2.80
A07_ID52	17046755-17047055	0.08	0.20	0.04	0.10	−	−	−	−
A07_S38	17079762-17079962	0.12	0.30	0.07	0.10	−	−	−	−
A07_S16	17171621-17171821	−	−	−	−	4.59	13.10	5.00	16.00
A07_S43	17310743-17310943	0.12	0.30	0.07	0.10	−	−	−	−
A07_S45	17399470-17399670	9.38	23.00	13.27	31.00	−	−	−	−
A07_S35	17538617-17538817	9.38	23.00	13.27	31.00	4.40	12.60	4.38	14.20
A07_S06	17601827-17602027	0.76	1.60	0.56	1.10	−	−	−	−
A07_S21	17804469-17804669	−	−	−	−	1.31	3.40	0.20	0.60

^a^ Physical position of markers based on *Brassica rapa* V1.5. ^b^ Explains phenotypic variation (%) in the segregating population. RD: root diameter; RW: root weight; LOD: likelihood of odd; ”−“: No data was shown.

**Table 3:** QTL mapping analysis of the *FR1.1* locus with the polymorphic markers.

Primer Name	Physical Position (bp)	RD	
		LOD Value	%Exp
A01_ID03	A01_4623486-4623786	0.86	1.90
A01_ID27	A01_4798250-4798550	6.22	15.40
A01_ID04	A01_4831809-4832109	7.01	17.20
A01_ID31	A01_5291260-5291560	0.28	0.60

RD: root diameter; LOD: likelihood of odd; %Exp: Explains phenotypic variation (%) in the segregating population.

## Data Availability

Data is contained within the article or supplementary material.

## Supplementary Materials

The following are available online at https://www.mdpi.com/1422-0067/22/2/653/s1. Figure S1. Sequence alignment of *Bra003652* and *AT1G78240* genes, Figure S2. Alignment of protein sequences of *Bra003652* and homologous genes from *Arabidopsis*, Chiifu, P2, P1 and radish. Black arrows indicate the positions of the amino acid variations between P2 and P1. Black lines indicate the S-adenosyl-L-methionine-dependent methyltransferase domain, Figure S3. Coding sequences alignment of *Bra003649* from both parents, Figure S4. Protein sequences alignment of *Bra003649* from both parents, Figure S5. Coding sequences alignment of *Bra003606* from parents and Chiifu, Figure S6. Protein sequences alignment of *Bra003606* from parents and Chiifu, Figure S7. Heatmap of showing correlation coefficient of pairwise comparison between traits. The number represents the correlation coefficient. Different colors represent different correlations. PW, plant width; PH, plant height; LW, leaf weight; RL, root length; RW, root weight; RD, root diameter, Table S1. Summary of the paired-end sequencing results, Table S2. Numbers of SNP and InDel detected in two pools and parental lines, Table S3. A list of 44 pairs InDel markers for *FR1.1*, Table S4. A list of 39 pairs of SNP markers and 8 pairs of InDel markers for *FR7.1*, Table S5. Information of 31 genes in the 17.31-17.60 Mb region on chromosome 7, Table S6. Candidate genes for swollen root formation in *B. rapa*, Table S7. A list of special primers used in qRT-PCR.

## Abbreviations

QTL	Quantitative trait loci
LOD	Likelihood of odd
qRT-PCR	Quantitative real-time polymerase chain reaction
SNP	Single nucleotide polymorphisms
InDel	Insertion and deletion
DNA	Deoxyribonucleic acid
RNA	Ribonucleic acid

## References

[1] Shattuck V.I., Kakuda Y., Shelp B.J., Kakuda N. (1991). Chemical composition of turnip roots stored or intermittently grown at low temperature. J. Am. Soc. Hortic. Sci..

[2] Gupta A.K., Singh J., Kaur N. (2001). Sink development, sucrose metabolising enzymes and carbohydrate status in turnip (*Brassica rapa* L.). Acta Physiol. Plant..

[3] Lu G., Cao J., Yu X., Xiang X., Chen H. (2008). Mapping QTLs for root morphological traits in *Brassica rapa* L. based on AFLP and RAPD markers. J. Appl. Genet..

[4] Vogl C.R., Reiner H., Vogl-Lukasser B.J.E.R. (2007). The Turnip (*Brassica rapa* L. subsp. *rapa*) in Eastern Tyrol (Lienz district; Austria). Ethnobot. Res. Appl..

[5] Zhang N., Zhao J., Lens F., de Visser J., Menamo T., Fang W., Xiao D., Bucher J., Basnet R.K., Lin K. (2014). Morphology, carbohydrate composition and vernalization response in a genetically diverse collection of Asian and European turnips (*Brassica rapa* subsp. *rapa*). PLoS ONE.

[6] Liu M., Bassetti N., Petrasch S., Zhang N., Bucher J., Shen S., Zhao J., Bonnema G. (2019). What makes turnips: Anatomy, physiology and transcriptome during early stages of its hypocotyl-tuber development. Hortic. Res..

[7] Kubo N., Saito M., Tsukazaki H., Kondo T., Matsumoto S., Hirai M. (2010). Detection of quantitative trait loci controlling morphological traits in *Brassica rapa* L.. Breed. Sci..

[8] Lou P., Zhao J., Kim J.S., Shen S., Del Carpio D.P., Song X., Jin M., Vreugdenhil D., Wang X., Koornneef M. (2007). Quantitative trait loci for flowering time and morphological traits in multiple populations of *Brassica rapa*. J. Exp. Bot..

[9] Wang X., Wang H., Wang J., Sun R., Wu J., Liu S., Bai Y., Mun J.-H., Bancroft I., Cheng F. (2011). The genome of the mesopolyploid crop species *Brassica rapa*. Nat. Genet..

[10] Witzel K., Neugart S., Ruppel S., Schreiner M., Wiesner M., Baldermann S. (2015). Recent progress in the use of ‘omics technologies in brassicaceous vegetables. Front. Plant Sci..

[11] Cheng F., Sun R., Hou X., Zheng H., Zhang F., Zhang Y., Liu B., Liang J., Zhuang M., Liu Y. (2016). Subgenome parallel selection is associated with morphotype diversification and convergent crop domestication in *Brassica rapa* and *Brassica oleracea*. Nat. Genet..

[12] Zhou B., Fan P., Li Y. (2014). High-throughput sequence analysis of small RNAs in skotomorphogenic seedlings of *Brassica rapa* ssp. *rapa*. Gene.

[13] Hearn D.J., O’Brien P., Poulsen T.M. (2018). Comparative transcriptomics reveals shared gene expression changes during independent evolutionary origins of stem and hypocotyl/root tubers in *Brassica* (Brassicaceae). PLoS ONE.

[14] Usuda H., Demura T., Shimogawara K., Fukuda H. (1999). Development of sink capacity of the “Storage Root” in a radish cultivar with a high ratio of “Storage Root” to shoot. Plant Cell Physiol..

[15] Luo X., Huang Q. (2011). Relationships between leaf and stem soluble sugar content and tuberous root starch accumulation in cassava. J. Agric. Sci..

[16] Rouhier H., Usuda H. (2001). Spatial and temporal distribution of sucrose synthase in the radish hypocotyl in relation to thickening growth. Plant Cell Physiol..

[17] Mitsui Y., Shimomura M., Komatsu K., Namiki N., Shibata-Hatta M., Imai M., Katayose Y., Mukai Y., Kanamori H., Kurita K. (2015). The radish genome and comprehensive gene expression profile of tuberous root formation and development. Sci. Rep..

[18] Fan M., Liu Z., Zhou L., Lin T., Liu Y., Luo L. (2010). Effects of plant growth regulators and saccharide on in vitro plant and tuberous root regeneration of cassava (*Manihot esculenta* Crantz). J. Plant Growth Regul..

[19] Wang Q., Zhang L., Guan Y., Wang Z. (2006). Endogenous hormone concentration in developing tuberous roots of different sweet potato genotypes. Agric. Sci. China.

[20] Roumeliotis E., Kloosterman B., Oortwijn M., Lange T., Visser R.G., Bachem C.W. (2013). Down regulation of *StGA3ox* genes in potato results in altered GA content and affect plant and tuber growth characteristics. J. Plant Physiol..

[21] Gao J., Cao X., Shi S., Ma Y., Wang K., Liu S., Chen D., Chen Q., Ma H. (2016). Genome-wide survey of Aux/IAA gene family members in potato (*Solanum tuberosum*): Identification, expression analysis, and evaluation of their roles in tuber development. Biochem. Biophys. Res. Commun..

[22] Koda Y., Ohkawa-Takahashi K., Kikuta Y. (2015). Stimulation of root thickening and inhibition of bolting by jasmonic acid in beet plants. Plant Prod. Sci..

[23] Jackson S.D. (1999). Multiple signaling pathways control tuber induction in potato. Plant Physiol..

[24] Hannapel D.J., Sharma P., Lin T., Banerjee A.K. (2017). The multiple signals that control tuber formation. Plant Physiol..

[25] Kumar D., Wareing P.F. (1973). Studies on tuberization in *Solanum andigena*: I. Evidence for the existence and movement of a specific tuberization stimulus. New Phytol..

[26] Xie Y., Xu L., Wang Y., Fan L., Chen Y., Tang M., Luo X., Liu L. (2018). Comparative proteomic analysis provides insight into a complex regulatory network of taproot formation in radish (*Raphanus sativus* L.). Hortic. Res..

[27] Yu R., Wang Y., Xu L., Zhu X., Zhang W., Wang R., Gong Y., Limera C., Liu L. (2015). Transcriptome profiling of root microRNAs reveals novel insights into taproot thickening in radish (*Raphanus sativus* L.). BMC Plant. Biol..

[28] Aksenova N.P., Konstantinova T.N., Golyanovskaya S.A., Sergeeva L.I., Romanov G.A. (2012). Hormonal regulation of tuber formation in potato plants. Russ. J. Plant Physiol..

[29] Zhang L., Li Z., Garraway J., Cai Q., Zhou Y., Li X., Hu Z., Zhang M., Yang J. (2020). The casein kinase 2 beta subunit CK2B1 is required for swollen stem formation via cell cycle control in vegetable *Brassica juncea*. Plant J..

[30] Krupkova E., Immerzeel P., Pauly M., Schmulling T. (2007). The *TUMOROUS SHOOT DEVELOPMENT2* gene of *Arabidopsis* encoding a putative methyltransferase is required for cell adhesion and co-ordinated plant development. Plant J..

[31] Qin Y., Cheng P., Cheng Y., Feng Y., Huang D., Huang T., Song X., Ying J. (2018). QTL-Seq identified a major QTL for grain length and weight in rice using near isogenic F2 population. Rice Sci..

[32] Shu J., Liu Y., Zhang L., Li Z., Fang Z., Yang L., Zhuang M., Zhang Y., Lv H. (2018). QTL-seq for rapid identification of candidate genes for flowering time in broccoli × cabbage. Theor. Appl. Genet..

[33] Takagi H., Abe A., Yoshida K., Kosugi S., Natsume S., Mitsuoka C., Uemura A., Utsushi H., Tamiru M., Takuno S. (2013). QTL-seq: Rapid mapping of quantitative trait loci in rice by whole genome resequencing of DNA from two bulked populations. Plant J..

[34] Li G., Chen H., Liu J., Luo W., Xie D., Luo S., Wu T., Akram W., Zhong Y. (2019). A high-density genetic map developed by specific-locus amplified fragment (SLAF) sequencing and identification of a locus controlling anthocyanin pigmentation in stalk of Zicaitai (*Brassica rapa* L. ssp. *chinensis* var. *purpurea*). BMC Genom..

[35] Semagn K., Babu R., Hearne S., Olsen M.J.M.B. (2014). Single nucleotide polymorphism genotyping using Kompetitive Allele Specific PCR (KASP): Overview of the technology and its application in crop improvement. Mol. Breed..

[36] Noh S.A., Lee H.S., Huh E.J., Huh G.H., Paek K.H., Shin J.S., Bae J.M. (2010). SRD1 is involved in the auxin-mediated initial thickening growth of storage root by enhancing proliferation of metaxylem and cambium cells in sweetpotato (*Ipomoea batatas*). J. Exp. Bot..

[37] Qu L., Wu C., Zhang F., Wu Y., Fang C., Jin C., Liu X., Luo J. (2016). Rice putative methyltransferase gene *OsTSD2* is required for root development involving pectin modification. J. Exp. Bot..

[38] Kaur I., Singh R., Singh D. (2017). Correlation and path coefficient analysis for yield components and quality traits in radish (*Raphanus sativus* L.). Agric. Res. J..

[39] Gurmu F., Shimelis H.A., Laing M.D. (2017). Correlation and path-coefficient analyses of root yield and related traits among selected sweetpotato genotypes. S. Afr. J. Plant Soil..

[40] Murray M.G., Thompson W.F. (1980). Rapid isolation of high molecular weight plant DNA. Nucleic Acids Res..

[41] Dai M., Thompson R.C., Maher C., Contreras-Galindo R., Kaplan M.H., Markovitz D.M., Omenn G., Meng F. (2010). NGSQC: Cross-platform quality analysis pipeline for deep sequencing data. BMC Genomics.

[42] Li H., Durbin R. (2009). Fast and accurate short read alignment with Burrows-Wheeler transform. Bioinformatics.

[43] Abe A., Kosugi S., Yoshida K., Natsume S., Takagi H., Kanzaki H., Matsumura H., Yoshida K., Mitsuoka C., Tamiru M. (2012). Genome sequencing reveals agronomically important loci in rice using MutMap. Nat. Biotechnol..

[44] Van Ooijen J.W., van’t Verlaat J.W., van Tol J., Dalen J., Buren J., van der Meer J., van Krieken J.H., van Kessel J., Voorips R., van den Heuvel L. (2006). JoinMap 4.0: Software for the Calculation of Genetic Linkage Maps in Experimental Population.

[45] Van Ooijen J.W., Boer M.P., Jansen R.C., Maliepaard C. (2000). MapQTL 4.0: Software for the calculation of QTL positions on genetic maps (user manual). Plant Res. Int..

[46] Xiao D., Zhang N., Zhao J., Bonnema G., Hou X. (2012). Validation of reference genes for real-time quantitative PCR normalisation in non-heading Chinese cabbage. Funct. Plant. Biol..

[47] Livak K.J., Schmittgen T.D. (2001). Analysis of relative gene expression data using real-time quantitative PCR and the 2(-Delta Delta C(T)) Method. Methods.

[48] (2000). Analysis of the genome sequence of the flowering plant *Arabidopsis thaliana*. Nature.

[49] Zhang X., Yue Z., Mei S., Qiu Y., Yang X., Chen X., Cheng F., Wu Z., Sun Y., Jing Y. (2015). A *de novo* genome of a Chinese radish cultivar. Hortic. Plant J..

